# Evolution of the Electromagnetic Manipulation: From Tunable to Programmable and Intelligent Metasurfaces

**DOI:** 10.3390/mi12080988

**Published:** 2021-08-20

**Authors:** Sisi Luo, Jianjiao Hao, Fuju Ye, Jiaxin Li, Ying Ruan, Haoyang Cui, Wenjun Liu, Lei Chen

**Affiliations:** 1 College of Electronics and Information Engineering, Shanghai University of Electric Power, Shanghai 200090, China; 19105093@mail.shiep.edu.cn (S.L.); y20205046@mail.shiep.edu.cn (J.H.); 20171695@mail.shiep.edu.cn (F.Y.); 1984221478@mail.shiep.edu.cn (J.L.); ying_ruan@126.com (Y.R.); haoyangcui@yeah.net (H.C.); 2 Finemade Microelectronics, Co., Ltd., Shenzhen 518000, China; liuwenjun@superchip.cn

**Keywords:** metamaterial, metasurface, electromagnetic manipulation, tunable metasurfaces, active programmable metasurfaces, intelligent metasurfaces

## Abstract

Looking back on the development of metamaterials in the past 20 years, metamaterials have gradually developed from three-dimensional complex electromagnetic structures to a two-dimensional metasurface with a low profile, during which a series of subversive achievements have been produced. The form of electromagnetic manipulation of the metasurface has evolved from passive to active tunable, programmable, and other dynamic and real-time controllable forms. In particular, the proposal of coding and programmable metasurfaces endows metasurfaces with new vitality. By describing metamaterials through binary code, the digital world and the physical world are connected, and the research of metasurfaces also steps into a new era of digitalization. However, the function switch of traditional programmable metamaterials cannot be achieved without human instruction and control. In order to achieve richer and more flexible function regulation and even higher level metasurface design, the intelligence of metamaterials is an important direction in its future development. In this paper, we review the development of tunable, programmable, and intelligent metasurfaces over the past 5 years, focusing on basic concepts, working principles, design methods, manufacturing, and experimental validation. Firstly, several manipulation modes of tunable metasurfaces are discussed; in particular, the metasurfaces based on temperature control, mechanical control, and electrical control are described in detail. It is demonstrated that the amplitude and phase responses can be flexibly manipulated by the tunable metasurfaces. Then, the concept, working principle, and design method of digital coding metasurfaces are briefly introduced. At the same time, we introduce the active programmable metasurfaces from the following aspects, such as structure, coding method, and three-dimensional far-field results, to show the excellent electromagnetic manipulation ability of programmable metasurfaces. Finally, the basic concepts and research status of intelligent metasurfaces are discussed in detail. Different from the previous programmable metamaterials, which must be controlled by human intervention, the new intelligent metamaterials control system will realize autonomous perception, autonomous decision-making, and even adaptive functional manipulation to a certain extent.

## 1. Introduction

As an emerging research product in the 21st century, or a new type of artificial composite functional material, metamaterials are subwavelength artificial composite structural materials, whose unit size is generally less than half of the working wavelength. The concept of electromagnetic (EM) metamaterials originated from a Russian paper published by Veselago [1], a scientist of the former Soviet Union, in 1967. Later, it was translated into English and published in 1968, and “metamaterials” gradually became known to the world. Veselago also proposed left-handed materials, which have negative dielectric constants and magnetic conductivity [2,3,4]. Although this breakthrough concept subverted people’s cognition of traditional electromagnetic materials, the theory received little attention at that time because such double-negative materials could not be obtained in nature, and it was difficult to be verified by experiments. Until 1996, Pendry proposed a structure [5] in which the metal wires were arranged periodically according to certain rules, and finally the material with negative dielectric constants was obtained. By adjusting the period and the radius of the metal wires, the plasma frequency can be reduced to the microwave range. Later, in 1999, he further proposed to nest the two open copper rings inside and outside [6], which was the split-ring resonator (SSR). When working near the resonant frequency of the SRR ring, it can exhibit negative magnetic conductivity. Based on these theories, in 2001, D. R. Smith et al. combined the two structures and designed them to make the two negative frequency bands coincide [7], producing the first artificial electromagnetic metamaterial. Generally, with three-dimensional structure, electromagnetic metamaterials have unique physical properties that traditional materials do not have, such as inverse Cherenkov radiation effect, negative refractive index [8,9,10,11], lens [12,13,14,15], cloaking [16,17,18,19,20,21], illusion devices [22,23], and so on. Today, metamaterials have developed into a multi-disciplinary and comprehensive research direction, whose research field is no longer limited to the electromagnetic field, but expanded to acoustics [13], thermal science [24], quantum mechanics [25], informatics [26], biomedical [27,28], and other disciplines, forming an extremely wide coverage and far-reaching important discipline. Metamaterials provide a wide space for people to manipulate electromagnetic waves [13] and even acoustic and mechanical waves [29] freely with their super freedom of design, and further give rise to new electromagnetic applications such as perfect imaging [30,31], holographic imaging [32,33,34,35,36], electromagnetic black hole [37], metamaterial lenses [12,38,39,40,41], and other EM designs with multiple functions [42,43].

The initial research work of electromagnetic metamaterials is based on electromagnetic resonant structures in three-dimensional form, which are usually composed of metal and its dielectric structure stacked on top of each other. Such structural design is extremely difficult in actual fabrication, so the structure verification of three-dimensional metamaterials adopts single-layer two-dimensional structure. In addition, the three-dimensional metamaterial structure also has many limitations in terms of material loss and working frequency band. Therefore, how to realize two-dimensional electromagnetic metamaterials, namely electromagnetic metasurface, has gradually become the focus of scientific attention. In 1999, Sievenpiper first proposed a high impedance surface similar to a mushroom-shaped structure [44]. This kind of magnetic tape gap structure is considered to be one of the early studies of the electromagnetic metasurface because of its periodic arrangement of subwavelength and effective suppression of specific surface wave patterns. Capasso’s team published a paper in the journal *Science* that proposed “generalized Snell’s law” in 2011 [45], which became an important turning point in the history of metasurface research. They used a V-shaped element to achieve reflective control of the geometric phase. By changing the opening angle and rotation angle of the V-shaped arms, the reflective phase can be covered by 360°. Based on this regulation of the abrupt phase of the surface, Capasso’s team showed that both the gradient phase distribution and the rotational phase distribution can be used to deflect the scattered beam and generate the vortex beam, respectively. Thanks to the new methods and ideas provided by the generalized Snell’s law for people to design electromagnetic metasurfaces, a large number of studies on the application of metasurfaces are emerging. With the advantages of excellent electromagnetic control ability, low profile, low loss, and easy processing, two-dimensional metasurface has been the leader in the research of metamaterials in the last ten years, which has stimulated a variety of functions and applications, such as holographic imaging [46,47,48], vortex beam [49,50,51,52,53], ultra-thin invisibility cloak [54], absorbers [55,56,57], Huygens metasurface [58,59], non-magnetic non-reciprocity metasurfaces [60,61,62], and so on.

Most of the early metasurfaces were passive structures. In order to explore and extend the dynamic tunable function of metasurfaces, active and tunable metasurfaces have been proposed successively [63]. Compared with the passive metasurface, the active metasurface usually has the advantages of a wide frequency band, large adjustable range, and low loss, which brings great vitality for the development of the metasurface. Gil’s team implemented a frequency-tunable filter by introducing a varactor diode into the open resonant ring [64]. Then, by filling the opening resonant gap with N-type silicon material containing light doping [65], Aloyse controlled the light with metamaterial. Later, some researchers used active devices to achieve tunable electrically controlled metamaterials and tunable magnetically controlled metamaterials [66]. The core idea of the active tunable metasurface is to load active devices on each element, and realize the functions of polarization conversion [67,68], beam scanning [69], multi-beam, and wave absorption [70] while keeping the physical structure of the unit unchanged. Active devices include a varactor diode, triode, sensor, and so on. At present, the regulation methods of the tunable metasurface mainly include mechanical control [71,72,73], electric control [74,75], temperature control [76], and light control [77]. Mechanical control is to manipulate the phase by adjusting the physical size or rotation angle. Additionally, the electronic devices commonly used in electrical control are: PIN diodes, varactor diodes, and MEMS switches. Compared with mechanical control, electric control has lower system complexity, more flexible regulation form, and stronger beam regulation ability. By adopting appropriate regulation mode, the active tunable metasurface can enlarge the manipulation range of the phase and polarization mode of electromagnetic wave in microwave frequency band, and plays an irreplaceable role in realizing arbitrary polarization and arbitrary beam control. At the same time, the combination of metasurface and tunable materials such as graphene can make great contributions to the progress of terahertz technology [78,79], visible light [54,80], and the infrared light field [11,81].

To explore the possible connection between metasurface and digital information, Engheta’s team put forward the concept of “digital metamaterial” in 2014 [82] and proposed that the discrete structural design method can be introduced into the design process of metamaterial. However, this concept is still limited to the digitization of equivalent medium parameters, so it is hard to realize, and no follow-up research has been carried out. Meanwhile, Cui Tie Jun proposed a new theory of digital coding programmable metasurface in 2014 [83], opening a new chapter in metasurface research. The core idea of digital coding metamaterials is to introduce digital binary code into the design of metamaterials. Furthermore, digital information is integrated into all aspects of the design of metamaterials [84], such as structure, electromagnetic parameters, and functions. Since then, diverse EM functional designs in passive coding metasurface design has been proposed, such as holography [85,86], full-space control [87], acoustic field modulation [88], optically transparent metasurfaces [89,90], orbital angular momentum (OAM) beams [91], and multi-frequency manipulation [92,93]. However, due to the functional solidification of passive coding metasurface, its application scenarios and practical value are greatly limited [94]. Active programmable coding metamaterials are the inevitable direction of passive structure function extension. So far, plenty of active programmable metasurfaces based on PIN diodes and varactors have emerged, and the coding form has gradually expanded from the programmable phase [95,96] to programmable amplitude [97] and polarization [98,99]. However, active control of programmable metamaterials still requires human intervention to change the control instructions or programs to achieve the switch of different electromagnetic characteristics [100], such as switching different phase coding states, different polarization coding states, etc. Therefore, the intelligent metamaterials will be an important direction in the future development of metamaterials [100,101,102,103].

This paper aims to review the evolution of electromagnetic manipulation: from tunable metasurfaces to active programmable metasurfaces and intelligent metasurfaces. With the rapid development of the metasurface, the design of tunable metasurface structure has changed from simple to complex, and the functions it can present have transformed from single to diverse. Moreover, the proposal of coding metamaterials gives new ideas to metamaterials, and various kinds of programmable metamaterials have been designed. In addition, the dynamic programmable property of digital coding metamaterials endows a high degree of freedom for functional design. Programmable phase, amplitude, polarization, and other coding forms rapidly give birth to a series of real-time tunable electromagnetic applications. On this basis, the design of intelligent metamaterials is also flourishing, and the intelligent judgment and decision are realized in a real sense, which lays the foundation for the further development of intelligent metamaterials and the realization of cognitive metamaterials. We start the introduction from the basic concept, research status, and regulation mode of tunable metasurfaces in Section 2, as well as examples given to illustrate how the phase and amplitude response can be flexibly manipulated by the tunable metasurfaces based on mechanical, electrical, and temperature control. In Section 3, we introduce the concept and design method of the coding metasurface, and describe in detail the structure, coding method, and far-field results of the active programmable metasurface based on PIN diodes, active amplifiers, transistors, thermistors, and photoresistors. Finally, we focus on the intelligent metasurface and introduce a dual-polarization programmable metasurface with intelligent sensing function.

## 2. Tunable Metasurfaces

The manipulation characteristics of electromagnetic metamaterials to electromagnetic waves are closely related to the geometric parameters and material parameters of the microstructure. Therefore, once the microstructure with specific functions is designed and formed, the regulation function of the microstructure to electromagnetic waves cannot be adjusted, resulting in a waste of resources to a certain extent. In addition, the dynamic control of electromagnetic wave has a wide range of applications in beam shaping, laser detection, scanning focusing, polarization regulation, laser sensing detection, signal tuning, and so on. On the other hand, three-dimensional metamaterials face the problems of high processing difficulty and high ohmic loss. In recent years, two-dimensional metamaterials with sub-wavelength thickness (metasurface) have become a research hotspot due to their advantages of low loss and easy processing. Consequently, how to use the tunable metasurface to dynamically manipulate the electromagnetic wave has become a subject worth studying in the field of metamaterials.

**Figure 1:**
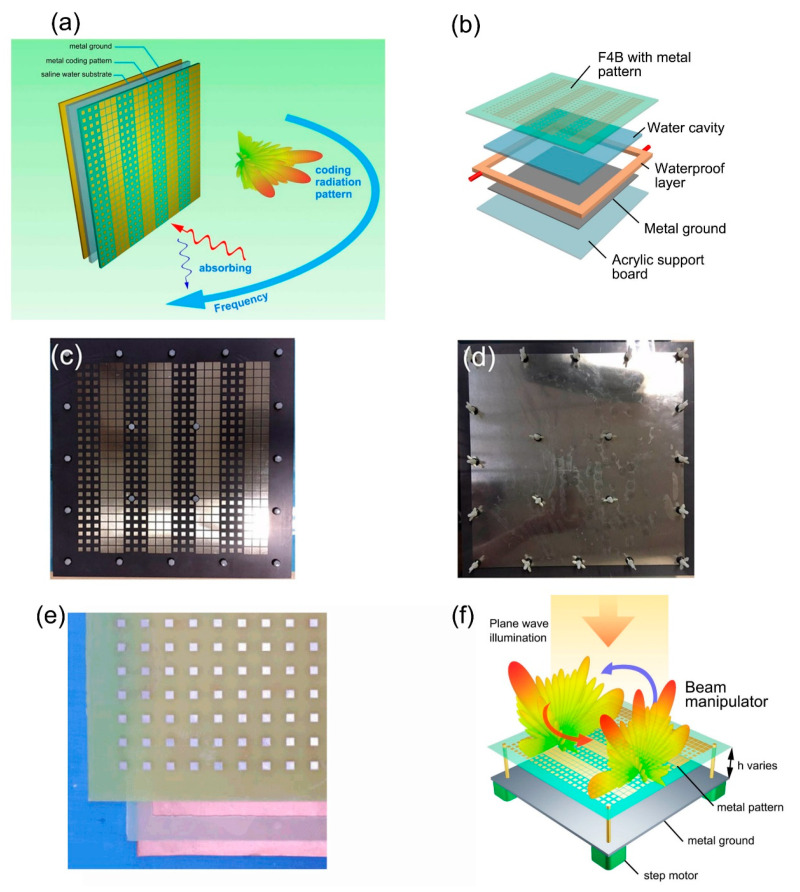
(**a**) The schematic of tunable metasurface based on saline water substrate (from Figure 1 of Ref. [104]). (**b**) The detailed structure of the fabricated sample (from Figure 8a of Ref. [104]). (**c**,**d**) The top and bottom views of the fabricated sample (from Figure 8c,d of Ref. [104]). (**e**) The schematic of preassembled tunable metasurface absorber based on temperature control (from Figure 4a of Ref. [105]) (**f**) Schematic diagram of reconfigurable metasurface based on mechanical control (from Figure 1 of Ref. [106]). Reproduced with permission from Ref. [104] under a Creative Commons Attribution 4.0 International License. Reproduced with permission from Ref. [105] provided by AIP Publishing and Copyright Clearance Center, license number: 5102270565341. Reproduced with permission from Ref. [106] provided by AIP Publishing and Copyright Clearance Center, license number: 5102240236980.

At present, many tunable metasurfaces based on mechanical control, temperature control, material attribute control, electric control, and light control have been designed. As shown in Figure 1a, the bifunctional tunable metasurface based on saline water consists of a substrate containing specific metal pattern [104], saline water substrate and metal ground from top to bottom. By transforming the concentration of the saline water substrate, the absorption performance of high frequency can be adjusted while keeping the low frequency scattering mode unchanged. Figure 1b is a detailed structure of the fabricated sample of the tunable metasurface. The top layer uses printed circuit board (PCB) technology to print a specific metal pattern on the F4B substrate, and the water cavity and waterproof layer are between the F4B substrate and the metal ground. The waterproof layer is made of PVC, which surrounds the whole cavity completely, and two small pipes are used to exchange water. The entire metasurface structure is supported by an acrylic board. The top and bottom views of the fabricated sample are shown in Figure 1c,d, respectively. Moreover, the measured results have good agreement with the simulation. Mechanical control mainly realizes phase and amplitude control by adjusting the physical size or rotation angle, while temperature control mainly alters electromagnetic performance through the sensitivity of dielectric substrate to temperature. The tunable water-substrate metasurface absorber is shown in Figure 1e, which modulates the absorption performance by controlling the environment temperature. It can be seen from Figure 1e that the metasurface absorber is a sandwich structure consisting of a patterned metal layer [105], a dielectric substrate, and metal ground from top to bottom. Unlike ordinary metal-substrate-metal metasurface absorbers, the proposed metasurface substrate is not only one substrate, but a mixture of water-based substrate and a low-permittivity material (LPM) substrate. The purpose is to allow water to be easily wrapped between the bottom metal ground and LPM layer, so that water can be used as the loss source of electromagnetic waves and thermal tuning environment, and the LPM layer can improve impedance matching and facilitate strong absorption. Figure 1f is a schematic diagram of the metasurface based on mechanical control [106]. It adopts flexible printed circuit (FPC) technology to print metal patterns on flexible polyester amide film, with an air substrate between the thin film and metal ground. The whole metasurface is fixed on four posts, and the thickness of the air substrate is controlled by precision stepper motors. Finally, our simulation data and measured data have a high degree of consistency.

**Figure 2:**
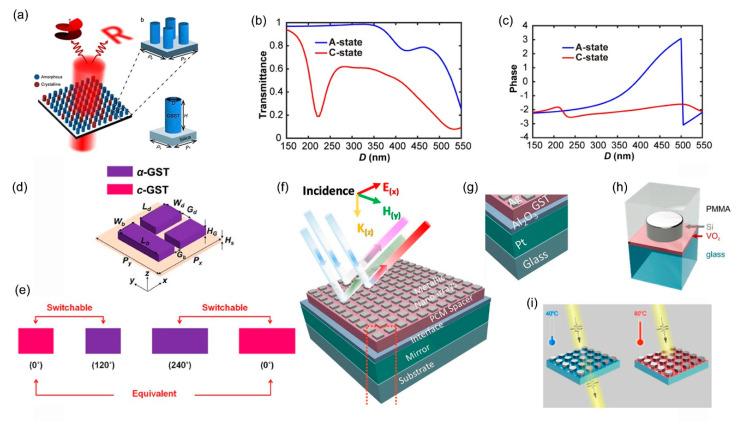
(**a**) The schematic of the tunable metasurface based on GSST (from Figure 1a–c of Ref. [107]). (**b**,**c**) The amplitude and phase responses of the metasurface. (from Figure 1d,e of Ref. [107]). (**d**) The schematic of an active dielectric metasurface based on GST (from Figure 4a of Ref. [108]). (**e**) Three different phase responses of 0°,120° and 240° (from Figure 5b of Ref. [108]). (**f**,**g**) Schematic diagram of a perfect metasurface absorber based on GST thin film (from Figure 1a,b of Ref. [109]). (**h**) The schematic of a tunable dielectric metasurface based on VO_2_ (from Figure 1a of Ref. [110]). (**i**) Two states of GST under different temperatures (from Figure 1b of Ref. [110]). Reproduced with permission from Ref. [107] under a Creative Commons Attribution 4.0 International License. Reproduced with permission from Ref. [108] under a Creative Commons Attribution-NonCommercial-NoDerivatives 4.0 International License. Reproduced with permission from Ref. [109] under a Creative Commons Attribution 4.0 International License. Reprinted (adapted) with permission from Ref. [110]. Copyright 2021 American Chemical Society.

Phase-change materials (PCMs) can greatly change the dielectric constant by transferring the lattice inside the material under external excitation (such as heat, laser, applied voltage). Therefore, PCMs provide a new method for dynamically regulating the optical properties of metasurface. Ge_2_Sb_2_Se_4_Te (GSST), Ge_2_Sb_2_Te_5_ (GST), and vanadium dioxide (VO_2_), as commonly used PCMs, have attracted extensive attention in the field of nano-photonics in recent years. GSST and GST both have two states: crystalline and amorphous, which can be converted to each other through temperature change. Figure 2a is a metasurface based on two states of GSST [107], where blue and red represent the amorphous and crystalline states, respectively. Each unit consists of a GSST nanopillar and a silicon substrate. As a dielectric resonator, the GSST nanopillar can perform amplitude and phase modulation in both crystalline and amorphous states, as shown in Figure 2b,c. It should be noted that phase response of the metasurface covers almost 360° in the amorphous state, but not in the crystalline state. Figure 2d shows an active dielectric metasurface based on GST [108]; rose and purple represent amorphous and crystalline states, respectively. As can be seen from Figure 2e, by changing the state and geometric parameters of GST, the metasurface can achieve three different phase responses of 0°,120°, and 240°. Figure 2f,g show a perfect metasurface absorber based on GST thin film [109], which adjusts GST between the crystalline and amorphous states through heat treatment, thus changing the visible light properties of PCM to achieve the reconfigurable metasurface. Unlike GSST and GST, VO2 has two stable states, both of which are crystalline states. Figure 2h is a tunable dielectric metasurface unit whose detailed structure is a silicon meta-atom embedded into glass surrounded by a VO2 layer [110]. When the temperature is lower than 68 °C, VO_2_ is a monoclinic crystal structure and is an insulator. Additionally, the VO_2_ phase changes into a tetragonal crystal structure and turns into a metal conductor when the temperature exceeds 68 °C. As shown in Figure 2i, the metasurface can achieve extinction function by using two states of GST under different temperatures. In summary, by combining PCMs, metasurface can better realize the manipulation of reflection amplitude and phase.

**Figure 3:**
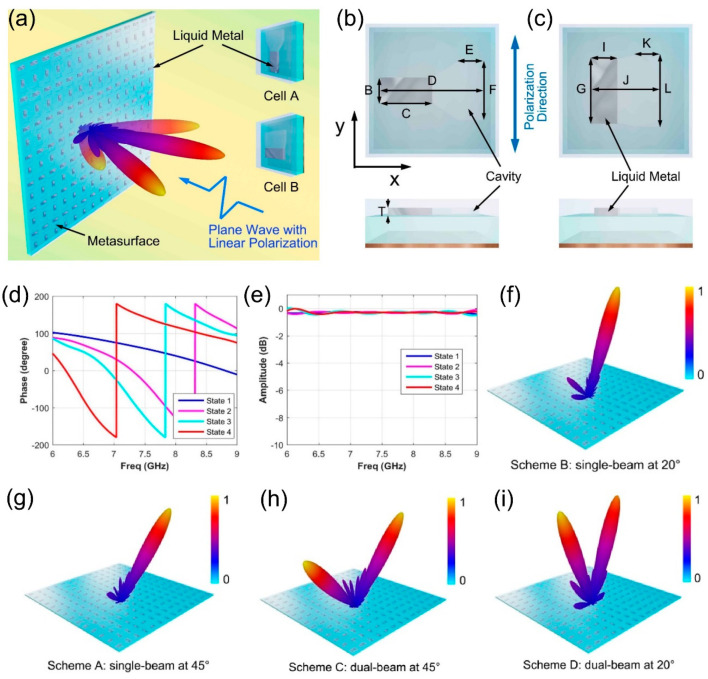
(**a**) The designed structure of the liquid metal metasurface for flexible beam-steering (from Figure 1 of Ref. [111]). (**b**,**c**) The top and side views of the cell A and cell B. (from Figure 2c,d of Ref. [111]). (**d**) Reflection phase responses of four different states composed of the cell A and cell B (from Figure 2e of Ref. [111]). (**e**) Reflection amplitude responses of four different states composed of the cell A and cell B (from Figure 2f of Ref. [111]). (**f**–**i**) The three-dimensional far-field results for arrays A, B, C, and D (from Figure 4a–d of Ref. [111]). Reproduced with permission from Ref. [111] under a Creative Commons Attribution 4.0 International License.

Many tunable materials in nature have been used in the design of tunable devices, such as transparent conductive oxides, two-dimensional materials (such as graphene, molybdenum disulfide), liquid crystals, semiconductors, and elastomers. The liquid metal metasurface for flexible beam-steering [111] is shown in Figure 3a, which consists of different metasurface elements A and B, on which cavities of different sizes are designed. At the same time, the liquid metal is injected into the cavity and filled into the desired structure by taking advantage of the characteristics of EGaln, which is easily affected by thermal stimulation and gravity. Figure 3b,c are, respectively, the top and side views of cell A and B, which are composed of acrylic substrate, FR4 substrate, and cooper ground. The cavity containing liquid metal serves as an interface between the acrylic substrate and the FR4 substrate. It is a multilateral structure with two rectangular cavities connected by a trapezoidal cavity in the middle. According to the marks in Figure 2b, the direction of the electric field, namely the direction of polarization, is along the *Y*-axis, while the reflection phase response is mainly affected by the length of the liquid metal patch in the direction of the electric field. Liquid metal is distributed in two rectangular cavities of cell A and B, so four liquid metals of different lengths, widths, and heights are designed in the rectangular cavity, and four different phase responses are realized under the condition that the volume of liquid metal is all 20 mm^3^. The detailed data of four states are 3 × 6.67 mm (B × C in cell A), 6.9 × 2.9 mm (F × E in cell A), 7.55 × 2.65 mm (G × I in cell B), and 8.5 × 2.35 mm (L × K in cell B). The reflected phase and amplitude responses of four different states of the metasurface element are shown in Figure 3d,e. At 7.5 GHz, the phase responses of the four states are 144.6°, 59.3°, −32.2°, and −124.4°, respectively. The phase difference between the two states is about 90°, and 360° phase coverage is achieved, showing good phase manipulation performance. Additionally, in the frequency band of 6–9 GHz, the amplitude responses of four different states are all close to 0 dB. The three dimensional far-field results of four different metasurface arrays at 7.5 GHz are depicted in Figure 3f–i. When the linearly polarized incidence plane wave illuminates the metasurface, by arranging four elements of different states to form diverse metasurface, the scattering fields of single and dual beams from different deflection angles can be realized. In conclusion, four kinds of metasurface elements with different phase response are designed by using the good fluidity and conductivity of EGaln, and the designed metasurface based on liquid metal can manipulate the deflection angle and amount of the beam.

**Figure 4:**
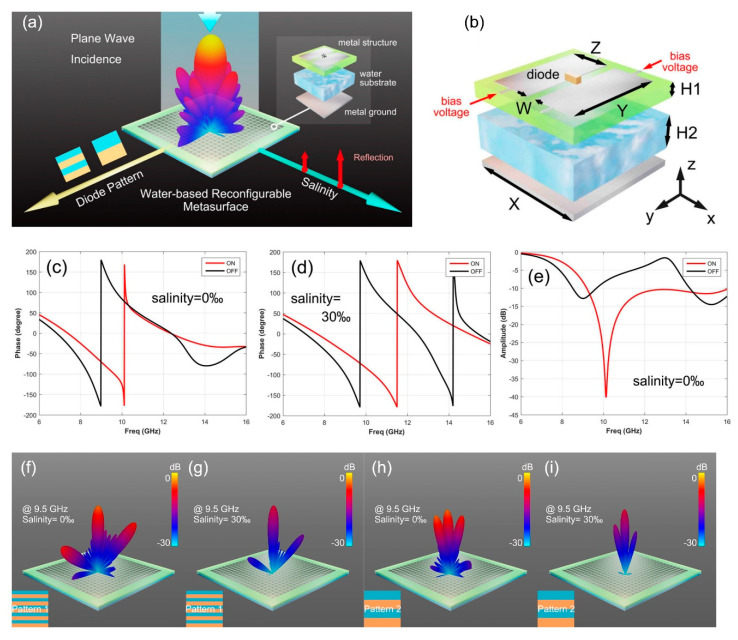
(**a**) Schematic of programmable metasurface based on water and integrated with PIN diodes (from Figure 1 of Ref. [112]). (**b**) Detailed structure of the metasurface element (from Figure 2a of Ref. [112]). (**c**) The reflected phase response of PIN diode switching state when salinity is 0% (from Figure 2c of Ref. [112]). (**d**) The reflected phase response of PIN diode switching state when salinity is 30% (from Figure 2d of Ref. [112]). (**e**) The reflected amplitude response of PIN diode switching state when salinity is 0% (from Figure 2e of Ref. [112]). (**f**,**g**) The three-dimensional far-field results of pattern 1 for salinity of 0% and 30% at 9.5 GHz (from Figure 6a,b of Ref. [112]). (**h**,**i**) The three-dimensional far-field results of pattern 1 for salinity of 0% and 30% at 9.5 GHz (from Figure 7a,b of Ref. [112]). Reproduced with permission from Ref. [112] provided by AIP Publishing and Copyright Clearance Center, license number: 5102290721661.

Compared with mechanical control, electric control has lower system complexity, more flexible regulation form and stronger beam regulation ability. The electronic devices commonly used in electrical control are PIN diodes, varactor diodes, and MEMS switches. The metasurface shown in Figure 4a integrates salinity control and electrical control [112]. By changing the salinity or by controlling the switch of the PIN diode, the phase response, scattering beam amplitude, and deflection angle can be independently manipulated. The detailed structure of the metasurface unit is shown in Figure 4b, which consists of metal patch, F4B substrate, water substrate, and metal ground from top to bottom. The PIN diode connects two metal patches on the top layer, each of which is connected to two slender metal wires. Bias voltage is transmitted through two thin metal wires to two metal patches to control the switching of the PIN diode. Figure 4c,d show the change of the reflected phase response when the salinity varies from 0% to 30%. The red and black lines represent the on and off states of the PIN diode, respectively. The phase difference ranges from −211° (diode on) to 90° (diode off) at 9.5 GHz with salinity at 0%, and from −4° (diode on) to −205° (diode off) at 10.5 GHz with salinity at 30%. This indicates that adjusting the salinity can significantly modulate the reflected phase response. As can be seen from the reflected amplitude response diagram in Figure 4e, the on and off states of the diode lead to different magnitudes of the reflected amplitudes, so the switching state of the diode can modulate the reflected amplitudes in a wide frequency range. In order to demonstrate the function of the proposed metasurface, two different metasurface patterns with 20 × 20 elements are designed. The two patterns are periodic, with pattern 1 consisting of two lines of on and off states diodes and pattern 2 composing of five lines of on and off state diodes. The three-dimension far-field results of patterns 1 and 2 at 9.5 GHz are shown in Figure 4f–i. All patterns produced three beams, and the beam deflection angle is about 19° for pattern 2 and about 51° for pattern 1. The reason is that the period of pattern 2 is larger than pattern 1. According to the generalized Snell’s law, large periods produce smaller deflection angles. Therefore, the reconfigurable water-based metasurface can modulate the reflected phase response by changing the salinity, and the scattering beam deflection angle can be controlled by arranging diodes in different switching states to form metasurface patterns. These two manipulations can be performed simultaneously and independently, allowing for a wider modulation range.

## 3. Active and Programmable Metasurfaces

Traditional research on metamaterials and metasurfaces is based on the continuous scale to design their electromagnetic characteristics. The analysis on the continuous scale can be summed up as analog, namely “analog metamaterials”. With the establishment and wide application of the von Neumann computer system, information representation cannot do without digital binary coding in modern information system. In 2014, the concept of digital metamaterial was first put forward, and then Cui Tie Jun’s research group proposed the idea of coding metamaterial, that is, using the state arrangement of “0” and “1” to regulate the electromagnetic wave. He also presented another important concept, “field programmable metamaterial”, which is to load active devices on each cell to form a programmable metasurface. The active devices include a varactor, triode, and sensor. Compared with the passive coding metasurface, the active field programmable metasurface is more flexible.

**Figure 5:**
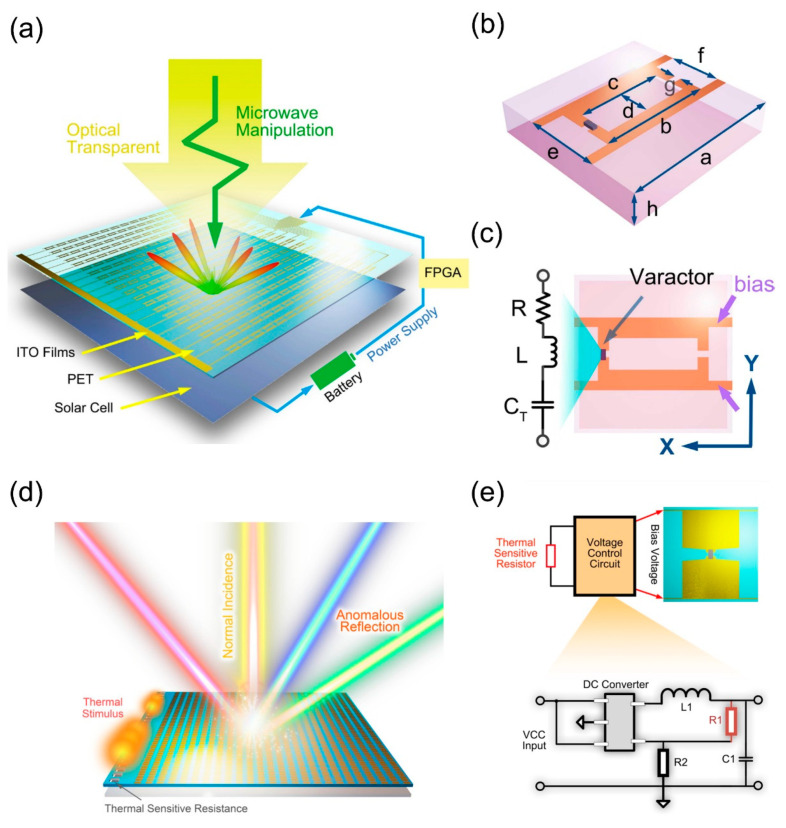
(**a**) The schematic of optical transparent and programmable metasurface integrated with varactor diodes (from Figure 1 of Ref. [113]). (**b**) Detailed structure of the metasurface element (from Figure 2a of Ref. [113]). (**c**) The equivalent RLC model of the varactor diode (from Figure 2b of Ref. [113]). (**d**) Schematic diagram of the programmable metasurface based on thermal sensor (from Figure 1 of Ref. [114]). (**e**) Detailed structure of the bias voltage control circuit. (from Figure 3a,b of Ref. [114]). Reproduced with permission from Ref. [113] provided by IOP Publishing and Copyright Clearance Center, license number: 1130743-1. Reproduced with permission from Ref. [114] provided by AIP Publishing and Copyright Clearance Center, license number: 5102300890023.

As shown in Figure 5a, the coding metasurface based on a varactor is composed of indium tin oxide (ITO) film, PET substrate, and solar cells from top to bottom [113]. By loading the varactor on each unit cell and changing the capacitances of the varactor, the reflected phase response of metasurface can be modulated. In addition, the elements integrate the varactors with different capacitances, which generate the diverse phase responses, are encoded into various digital states. By arranging these digital states into different coding sequences, the scattering field beam can be manipulated. The voltage of the varactor is provided by a solar battery, and the state of the varactor is controlled flexibly by FPGA. The detailed structure of the designed unit cell is shown in Figure 5b. The top layer is two symmetrical ITO films connected by a varactor diode, and the PET substrate is located between the top layer ITO film and the bottom layer ITO film. In general, the varactor diode can be regarded as a series model of RLC, and its equivalent circuit is shown in Figure 5c. Three elements with different capacitances, and thus different phase responses, are encoded as digital codes “1”, “2”, and “4”, respectively. In addition, we have also obtained the measured results which are in agreement with the simulation results. The combination of sensor and metasurface to manipulate electromagnetic waves is one of the research hotspots in recent years. As shown in Figure 5d, the programmable metasurface based on thermal sensor consists of an array of thermistors and 1-bit programmable metasurface array [114]. When the temperature changes, the resistance of the thermistor transforms, thus affecting the bias voltage value. Then, the switching of the PIN diode state gives rise to the modulation in reflective phase responses of the unit cells. As depicted in Figure 5e, the top layer of the unit cell is composed of two symmetrical metal patches, and both ends of the PIN diode (Skyworks SMP1320) are connected to the two metal patches. The voltage is transmitted through the bias line to the metal patch to control the switching state of the PIN diode. The resistance R1 marked in red is a thermistor, and its resistance will vary with the change of temperature. Finally, the results of the simulation and test show a high degree of consistency.

**Figure 6:**
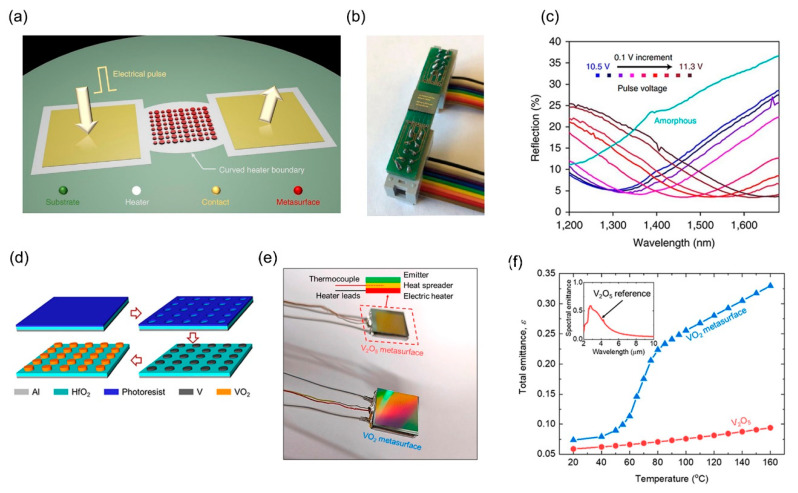
(**a**) The schematic of an electrically reconfigurable non-volatile metasurface based on GSST (from Figure 1a of Ref. [115]). (**b**) The metasurface is connected to a printed circuit board (PCB) carrier by wire (from Figure 1b of Ref. [115]). (**c**) The reflection response of the metasurface at different pulse voltages (from Figure 4a of Ref. [115]). (**d**) Schematic diagram of the VO_2_-based metasurface (from Figure 1a of Ref. [116]). (**e**) A diagram of the metasurface sample based on V_2_O_5_ and VO_2_ (from Figure 4a of Ref. [116]). (**f**) The total emittance of the tunable VO_2_ metasurface emitter and V_2_O_5_ metasurface emitter (from Figure 4b of Ref. [116]). Reprinted (adapted) with permission from Ref. [117]. Copyright 2021 American Chemical Society.

GSST has a large non-volatile exponential modulation capability, broadband low optical loss, and large reversible switching capacity, which enables the active metasurface to be continuously tunable. Figure 6a is an electrically reconfigurable non-volatile metasurface based on GSST [115]. Electrically reconfigurable metasurfaces are placed on a metal heater, and meta-atoms are patterned in a GSST film. The crystalline and amorphous states of GSST are controlled by pulse voltage. When the pulse voltage is long and low, joule heating triggers crystallization and GSST is in the crystalline state. When the pulse is short and high pressure, meta-atoms melt, are quenched, and recover, and GSST is in an amorphous state. The metasurface is connected to a printed circuit board (PCB) carrier by wire, as shown in Figure 6b. Figure 6c is the reflection response of the metasurface at different pulse voltages, which realizes the broadband tuning of the metasurface. The absorption loss of VO_2_ in the infrared band is low, so the VO_2_-based metasurface shown in Figure 6d can realize thermally switching the excitation of magnetic polariton to enhance infrared emission [116]. The metasurface is composed of VO_2_, HfO_2_ substrate, photoresist substrate, and Al substrate from top to bottom. When the temperature exceeds the phase transition temperature, the magnetized pole is excited by VO_2_, and the tunable metasurface achieves the enhancement of thermal emission. Figure 6e is a diagram of the metasurface sample based on V_2_O_5_ and VO_2_. The total emittance of the tunable VO_2_ metasurface emitter and V_2_O_5_ metasurface emitter is shown in Figure 6f. VO_2_ tunable metasurface can be regarded as a diffuse infrared emitter because of phase transition with temperature increase. However, V_2_O_5_ metasurface does not undergo phase transformation, and its emissivity is comparable to that of the insulated VO_2_ metasurface before phase transformation. Therefore, the combination of metasurface and PCMs can realize the regulation of electromagnetic waves.

**Figure 7:**
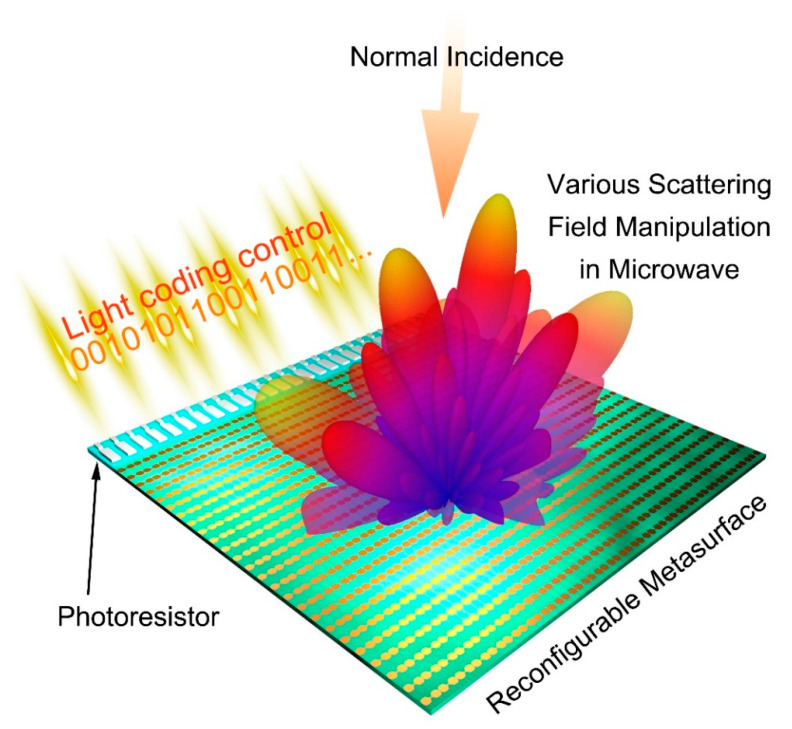
Schematic diagram of the optically controlled coding metasurface (from Figure 1 of Ref. [117]). Reproduced with permission from Ref. [117] under a Creative Commons Attribution 4.0 International License.

**Figure 8:**
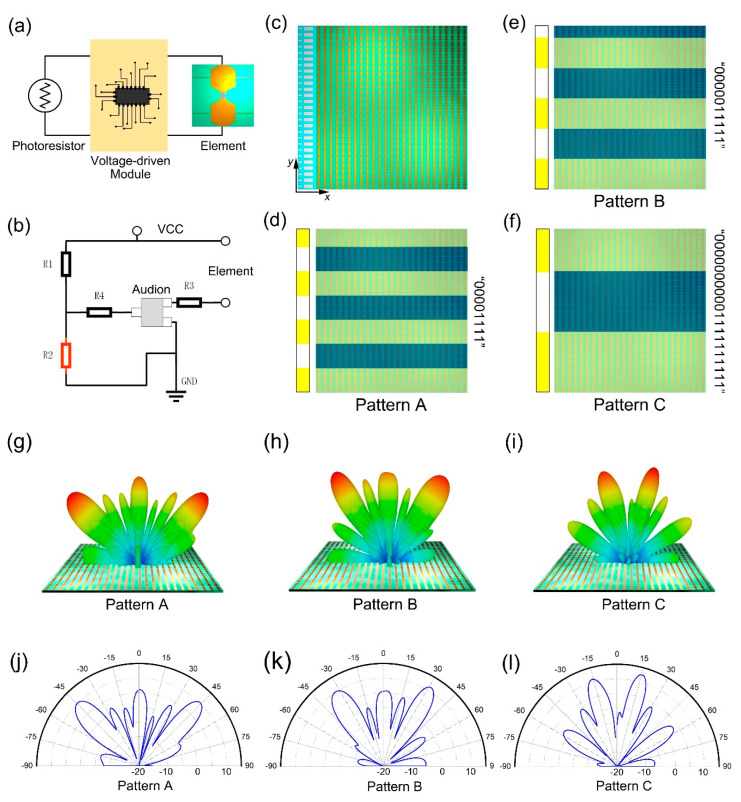
(**a**) Diagram of connection between photoresistor and element (from Figure 3a of Ref. [117]). (**b**) The voltage control circuit (from Figure 3b of Ref. [117]). (**c**) A schematic diagram of the metasurface consisting of 25 × 27 cells (from Figure 3c of Ref. [117]). The pattern with the coding sequence of (**d**) “00001111”, (**e**) “0000011111”, and (**f**) “00000000001111111111” (from Figure 3e,f of Ref. [117]). (**g**,**j**) The simulated far-field results pattern A. (from Figure 4a,b of Ref. [117]). (**h**,**k**) The simulated far-field results pattern B (from Figure 4c,d of Ref. [117]). (**i**,**l**) The simulated far-field results pattern C (from Figure 4e,f of Ref. [117]). Reproduced with permission from Ref. [117] under a Creative Commons Attribution 4.0 International License.

The metasurface in Figure 5d,e uses a thermistor to control the bias voltage in real time. Similarly, a photosensitive resistor can be applied to switch diode states. Figure 7 is a schematic diagram of an optically controlled coding metasurface [117], whose working mechanism is similar to the temperature-controlled coding metasurface. The photosensitive resistor is used to detect the change of the optical signal, and it is connected with a row of diode-loaded metasurface units through the voltage control module. The state of PIN diode can be switched by transforming the resistance of the photoresistor, thus modulating the reflection phase response. Therefore, by controlling the distribution of light, the metasurface can convert the coding sequence to produce different scattering fields as needed. Figure 8 shows the voltage control circuit and three specific coding sequences of the optically controlled metasurface. It should be noted that the digital code “0” and “1” states correspond to the diode on and off states, respectively. In combination with Figure 8a,b, the photoresistor alters with the change in light signal, transforming the optical signal into an electrical signal. The voltage control circuit has one end connected to a photosensitive resistor, so it feeds this transition back to the metasurface element and changes the state of the PIN diode. The photoresistor (R2) marked in red, and its resistance can be varied while all other resistors are fixed. Figure 8c is a schematic diagram of the metasurface consisting of 25 × 27 cells, each row of which is controlled by a photoresistor, so that their diode states are the same. Figure 8e,f are the metasurfaces of three different coding sequences. The coding sequences of pattern A, B and C are “00001111”, “0000011111”, and “000000000011111111”, respectively, and their simulated far-field results at 5.6 GHz are shown in Figure 8g–i. Patterns A and B both generated three beams with deflection angles θ = ±45° and θ = ±31°, respectively. According to the generalized Snell’s law, a larger period sequence produces a smaller deflection angle, so the deflection angle of pattern B is smaller than that of pattern A. Pattern C generates a double beam with deflection angle θ = ±15°. By designing the specific phase distribution on the metasurface, that is, designing the distribution of light, the scattering field of the metasurface can realize different electromagnetic scattering characteristics such as double beam or multiple beams.

**Figure 9:**
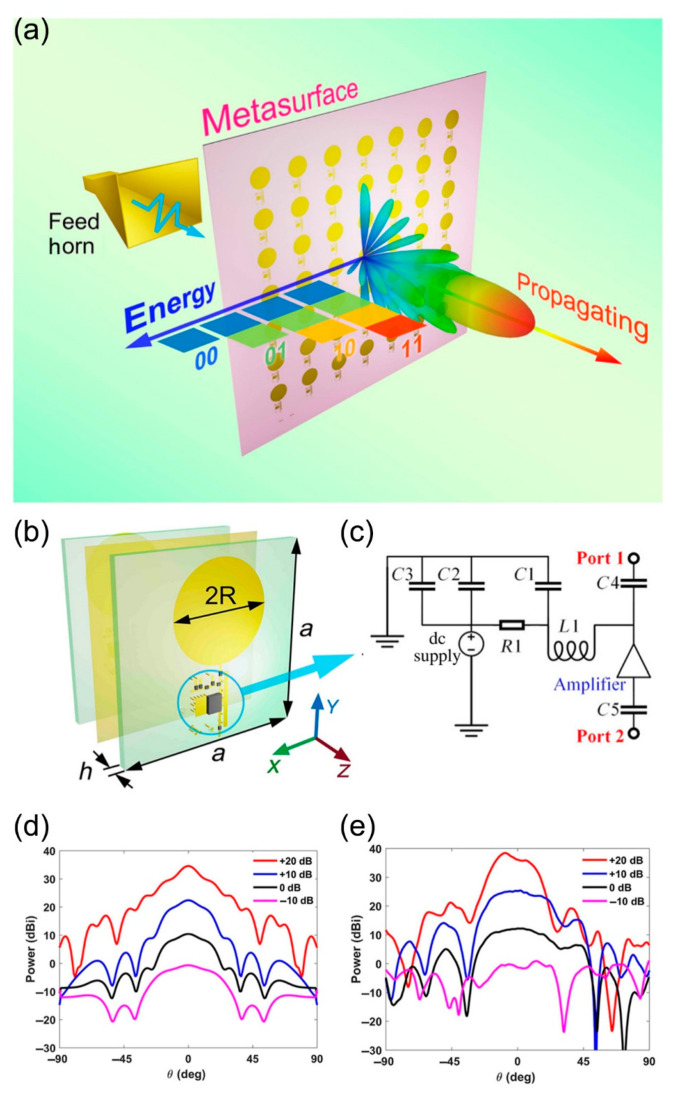
(**a**) Schematic of a spatial-energy digital-coding metasurface based on active amplifiers (from Figure 1 of Ref. [97]). (**b**) The detailed structure of the metasurface element (from Figure 2a of Ref. [97]). (**c**) The detailed circuit connections of its circuit components (from Figure 2b of Ref. [97]). (**d**,**e**)The simulated and measured far-field results of coding states “00”, “01”, “10”, and “11”(from Figure 7a,b of Ref. [97]). Reproduced with permission from Ref. [97] provided by the American Physical Society and SciPris. License number: RNP/21/JUL/041860.

In order to achieve richer and more flexible functional regulation and an even higher level of metasurface design, the coding programmable devices should not be limited to the consumption devices such as PIN diodes or varactors. Therefore, we need to study programmable metasurfaces based on transistor amplifiers and detectors. As shown in Figure 9a, a spatial-energy digital-coding metasurface based on active amplifiers is proposed [97]. By controlling the amplification level of the amplifier, the energy of the linearly polarized wave can be amplified or reduced. In other words, the energy of the space-propagated wave can be edited arbitrarily. In addition, by applying four specific voltages (3, 3.8, 4.2, and 5 V) to power amplifiers, the spatial energy of the propagating wave can be modulated to four different amplification levels (−10, 0, 10, and 20 dB). These specific amplification levels can be further encoded into digits such as “00,” “01,” “10,” and “11.” Figure 9b is the detailed structure of the metasurface element, which consists of two F4B substrates and a metallic ground located between the F4B layers. The metal structure of the top layer is mirrored to the structure of the bottom layer. At the same time, two power amplifier chips and necessary peripheral circuits are embedded in the top layer and the bottom layer, respectively. The amplification level of the amplifier chip can be adjusted by applying different supply voltages. The transmitted energy is first coupled to a circular metal patch at the top layer for amplitude modulation. Then, the coupling energy is amplified twice in two amplifiers and radiated into space through the circular metal patch at the bottom. The integrated amplifier circuit module is circled in blue, and the detailed circuit connections of its circuit components are shown in Figure 9c. Ports 1 and 2 in the module are connected to the circular metal patch and via hole, respectively. In Figure 9d,e, the simulated and measured far-field results of coding states “00”, “01”, “10”, and “11” are listed, respectively. At 5 GHz, the simulation of four different amplification levels agrees well with the measured far field results. In a word, by applying different supply voltages to change the amplifier’s amplification level, four different amplification levels from −10 dB to 20 dB can be realized. Furthermore, this work not only achieves the arbitrary manipulation of space-propagated wave energy, but also expands the application potential for active and programmable metasurface.

**Figure 10:**
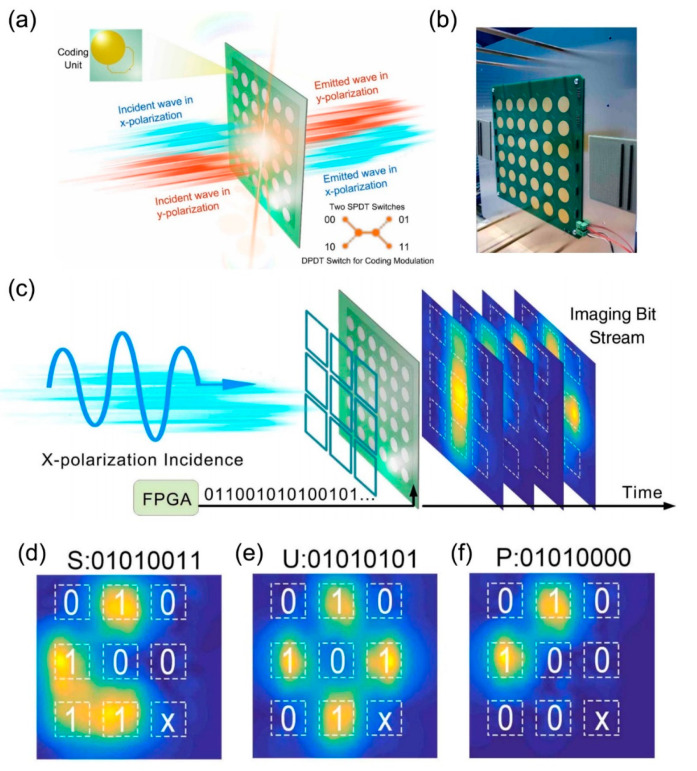
(**a**) Schematic of programmable metasurface based on SPDT switches (from Figure 1 of Ref. [98]). (**b**) The fabricated sample of the metasurface (from Figure 7a of Ref. [98]). (**c**) Schematic of the binary information representation and transmission (from Figure 6a of Ref. [98]) (**d**–**f**) The simulated near-field electric field distributions of three letters (SUP) with binary ASCII codes (from Figure 6f–i of Ref. [98]). Reproduced with permission from Ref. [98] under a Creative Commons Attribution 4.0 International License. Copyright: © 2021 Chinese Laser Press.

There are many coding metasurfaces designed for phase manipulation, but few programmable metasurfaces designed for polarization regulation. In Figure 10a, an active and programmable metasurface based on a single-pole double-throw (SPDT) switch is proposed [98]. The metasurface element is a double-sided metal patterned structure, with the top and bottom metal structures mirroring each other and both embedded with SPDT switches. The switching state of SPDT is controlled by a field programmable gate array (FPGA) which provides digital voltage. The multi-function polarization conversion can be realized by transforming the switching state of SPDT. When the metasurface is illuminated by a linearly polarized wave, four linear-to-linear polarization transmission states can be realized, such as X-to-X, X-to-Y, Y-to-X, and Y-to-Y. For the sake of simplicity, these four states can be encoded as “00”, “01”, “10”, and “11”. It should be noted that the digital code “0” and “1” states correspond to the x-polarized and y-polarized states, respectively. The fabricated sample of the metasurface, which is composed of 6*6 elements, is depicted in Figure 10b. Figure 10c is the schematic of the binary information representation and transmission. This work divides the metasurface into nine supercells, each of which contains 2*2 elements of the same transmission state. Under the illumination of the linearly polarized wave, the transmission energies of the nine supercells are not the same because of their different SPDT states. In order to better distinguish energy information, the low and high transmission energy can be encoded as digit “0” and “1”. In Figure 10d,e, the simulated near-field electric field distributions of three letters with binary ASCII codes are represented. Take the letter S of binary ASCII code 01010011 as an example to explain the transmission energy distribution diagram. It should be noted that X-to-X polarization and X-to-Y polarization are encoded as digit “1” and “0”. Therefore, the state of the first, third, fifth, and sixth supercells is set as X-to-Y polarization, and the state of the remaining four supercells is set as X-to-X polarization. As shown in Figure 10d, under the illumination of the x-polarized wave, the supercells which are encoded as “1” possess the high transmission energy. Similarly, the transmission energy distribution in Figure 10e,f can also correspond to the binary ASCII code information of the letter. Thus, by controlling the state of SPDT switch with FPGA, the programmable metasurface can not only convert transmitted electromagnetic waves between linear cross- and co-polarization, but also transmit binary coding sequences according to energy distribution.

## 4. Intelligent Metasurfaces

As an active and controllable form of coding metamaterials, programmable metasurfaces provide a hardware basis for the functional diversity of information metamaterials. A variety of programmable metasurfaces, such as phase, amplitude, and polarization programmable metasurfaces, have been proposed successively, showing the excellent electromagnetic manipulation ability of programmable metamaterials. However, at present, almost all programmable metasurfaces require human participation in the regulation of their electromagnetic characteristics or functions, that is, the operation of the control part needs to be carried out with the help of human subjective judgment and recognition. For intelligent metasurfaces, adaptive intelligent operation must make them have the ability to identify and judge the environmental changes actively [100,101], so as to make autonomous decisions according to certain intelligent algorithms.

**Figure 11:**
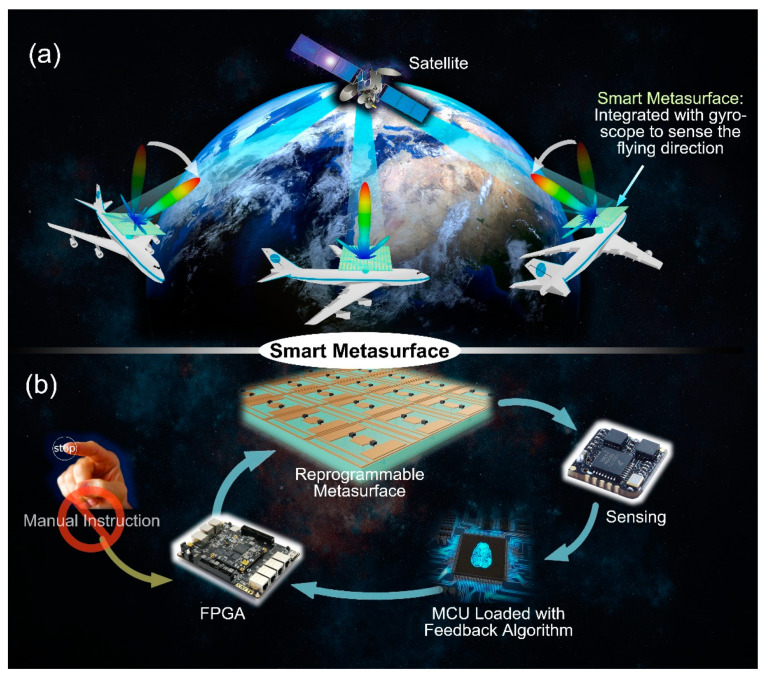
(**a**) The schematic diagram of the application scenario of metamaterial satellite communication on dynamic aircraft (from Figure 1a of Ref. [100]). (**b**) Intelligent metasurface control architecture: programmable metamaterials, sensing devices, intelligent feedback algorithms constitute a closed-loop decision loop (from Figure 1b of Ref. [100]). Reproduced with permission from Ref. [100] under a Creative Commons Attribution 4.0 International License. Copyright 2019, Springer Nature.

For this reason, this work assumed a special application scenario, as shown in Figure 11a. It is supposed that the reflected beam of a metasurface on a dynamic aircraft demands to be aligned adaptively to a satellite in real time for communication. When the aircraft is in different flight directions, the scattering angle of the electromagnetic beam scattered by the smart metasurface needs to be automatically aligned with the satellite in a fixed direction. In this control system, metasurfaces require to have the ability to detect the motion and attitude of the aircraft actively, as well as intelligent algorithms to process the perceptive data and make real-time decisions. Therefore, as depicted in Figure 11b, based on conventional programmable metasurfaces with programmable elements and control links, sensors are further added and a microcontroller unit (MCU) with intelligent feedback algorithm is loaded to form a closed-loop control loop [100]. Sensors on the metasurface can detect some certain characteristics of the metasurface and its environment (for example, spatial attitude, motion state, and temperature). In this control loop, the traditional programmable metasurface FPGA will no longer demand manual control, but directly through the MCU after some analysis and processing for intelligent manipulation. When the sensor detects the angle of aircraft attitude changes, the sensory data directly transmit to the MCU. Additionally, according to the change of attitude angle difference, as well as the set beam function (such as beam gaze communication), the MCU can calculate the required direction deflection quickly and generate a set of corresponding coding sequences of the metasurface. In this way, the complete adaptive closed-loop control is realized. This design idea makes the programmable metasurface possess the ability of intelligent judgment and decision in a real sense and provides a new idea for the intelligent development of the metasurface in the future.

**Figure 12:**
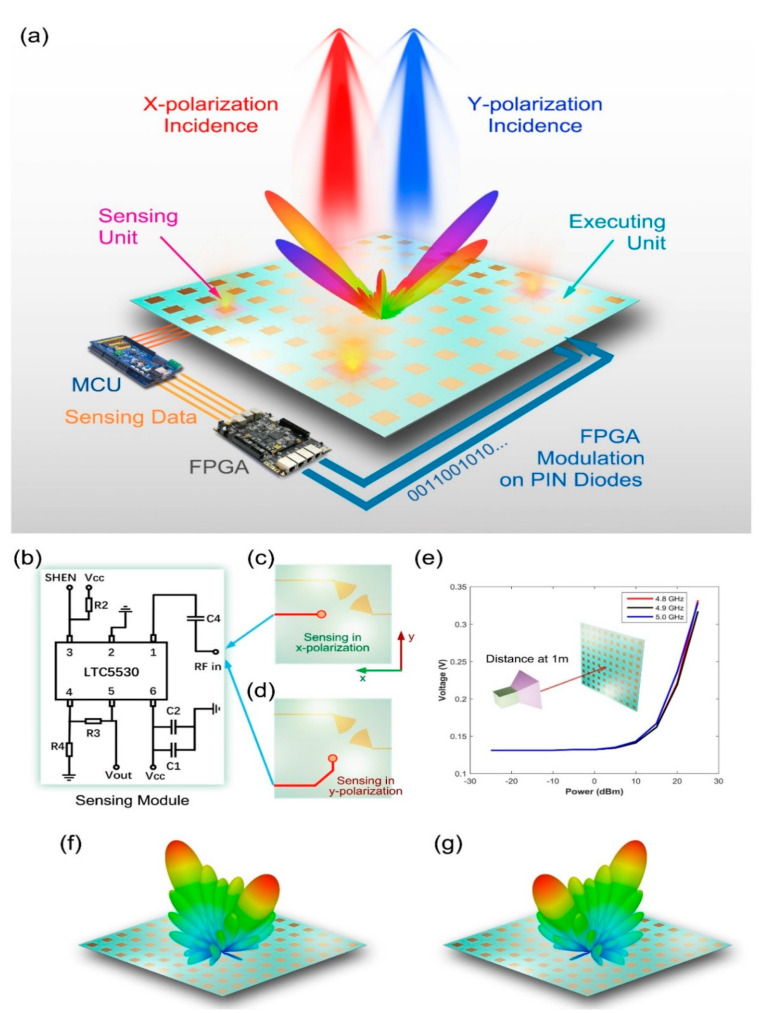
(**a**) Diagram of a dual-polarization programmable metasurface with intelligent sensing function (from Figure 1 of Ref. [101]). (**b**) The RF detection circuit module (from Figure 3a of Ref. [101]). (**c**,**d**) The bottom views of the sensing units for the x- and y-polarizations (from Figure 3a,c of Ref. [101]). (**e**) The test results of the three frequency points of 4.8 GHz, 4.9 GHz and 5.0 GHz (from Figure 3d of Ref. [101]). (**f**,**g**) The results of the dual-beam scattering field in the two polarization directions (x- and y-polarizations; from Figure 4b,c of Ref. [101]). Reproduced with permission from Ref. [101] under a Creative Commons Attribution 4.0 International License.

The intelligent metasurface shown in Figure 11 integrates sensors by a wire connection. Because the sensing device is separated from the programmable metasurface, and the metasurface cannot complete the sensing function, so the relative integration degree is low. Figure 12 introduces a dual-polarization programmable metasurface with intelligent sensing function [101], which can detect the polarization direction and energy of incidence wave actively and realize multi-functional intelligent beam manipulation. The metasurface consists of two types of units: the sensing unit and the executing unit. The executive unit is a common programmable metasurface unit, while the sensing unit has two functions of sensing and regulating electromagnetic waves. Under the illumination of different, polarized wave, the sensing units can obtain a DC voltage reflecting the incidence power level through the receiving circuit. After detecting the voltage, the MCU converts the analog voltage into a digital signal. Then, according to the preset control algorithm, it determines what kind of scattering field control instruction of the metasurface is adopted and transmits the instruction to the FPGA. Finally, the FPGA executes corresponding instructions to manipulate the diode voltage on the metasurface. As shown in Figure 12b, the RF detection circuit module is an important part of the sensing unit, which is composed of a commercial RF detection chip and peripheral circuit. Each sensing unit is equipped with such a group of sensing detection module circuit. At this point, the sensing unit can import the coupling energy of the surface metal patch through the hole to the back microstrip line connected with the RF detection circuit module, so as to realize the corresponding function. Figure 12c,d show the bottom views of the sensing units for the x- and y-polarizations. The microstrip lines of the x-polarization sensing unit are designed along the *x*-axis, while the microstrip lines of the y-polarization sensing unit extends along the *y*-axis and enters the detection circuit along the *x*-axis after a 45° broken line transition. This is designed to minimize the energy loss associated with turning corners. In order to verify the RF power detection performance of the sensing link, the output voltage of the detection circuit under different incidence wave power is tested. Figure 12e shows the test results of the three frequency points of 4.8 GHz, 4.9 GHz and 5.0 GHz. As can be seen from the diagram, when the input power of the horn antenna is greater than about 10 dBm, the output voltage increased obviously from 0.15 V to more than 0.3 V. The results of the dual-beam scattering field in the two polarization directions (x- and y-polarizations) are listed in Figure 12f,g. Two obvious scattering beams, respectively, direct to the x-and y-axes, and this verifies the coding design of double beam deflection.

## 5. Conclusions

In this paper, we review the recent five-year evolution of electromagnetic manipulation from tunable to programmable and intelligent metasurfaces. We mainly focus on illustrating tunable, active programmable, and intelligent metasurfaces from five aspects: basic concept, working principle, design method, manufacturing, and experimental verification. Looking back at the development trend of electromagnetic manipulation since it first attracted public attention, we can summarize several important stages. The first stage, research on electromagnetic manipulation, initially focused on passive metasurface design. The disadvantage of a passive metasurface is that once the metasurface is prepared, its function is fixed, which limits its application to some extent. Therefore, in order to realize the dynamic operation of electromagnetic waves, the form of electromagnetic manipulation evolves from passive to active tunable metasurface, which is the second stage. The core idea of tunable metasurface is to manipulate the electromagnetic wave without changing the physical structure of the element itself. At present, the regulation forms of the tunable metasurface include mechanical control, temperature control, light control, and so on. Great examples are given to illustrate the design methods of different tunable metasurfaces and how they can achieve flexible manipulation of phase and amplitude responses. In the third stage, with the emergence of the new theory of “digital coding programmable metasurface” a new chapter of metasurface electromagnetic manipulation has been initiated. The key idea of digitally encoding metamaterials is to introduce digital binary code into the design of metasurfaces and utilize the state arrangement of “0” and “1” to manipulate electromagnetic waves. We introduce active programmable metasurfaces based on a varactor, a triode amplifier, and a sensor. By encoding elements with different phases to form different coding sequences, the beam modulation of the scattering field is realized. In the fourth stage, electromagnetic manipulation has stepped into the adaptive and smart age, with the development of self-adaptively intelligent metasurface. Different from previous programmable metasurfaces, which must be controlled by human intervention, the new intelligent metasurface control system will realize autonomous perception, autonomous decision-making, and even adaptive functional control to a certain extent. For the future stage, because the digital coding metasurface needs to be controlled by FPGA to achieve a specific functional design, and the size of the FPGA cannot be ignored in practical application, which will inevitably affect the convenience and integration of the programmable metasurface. Therefore, whether the intelligent programmable metasurface can be separated from the FPGA and realize independent regulation of each element will be the potential direction to promote the development of intelligent metasurfaces.
